# Effect of Item Order on Certain Psychometric Properties: A Demonstration on a Cyberloafing Scale

**DOI:** 10.3389/fpsyg.2021.590545

**Published:** 2021-01-22

**Authors:** Murat Doğan Şahin

**Affiliations:** Department of Educational Sciences, Faculty of Education, Anadolu University, Eskişehir, Turkey

**Keywords:** item order effect, self-report questionnaire, modification indices, measurement invariance, cyberloafing

## Abstract

Many studies have been conducted on the effect of item order in self-report questionnaires on mean scores. This research aims to study the effect of item order on measurement invariance in addition to mean scores. To this end, two groups randomly obtained from the same sample were presented a fixed order form in which all items belonging to the same dimension were adjacent to each other, and a random order form in which the items were randomly sequenced respectively. The results obtained revealed a statistically significant difference between the mean scores of the two forms. In the next stage of the study, the fit indices obtained from the confirmatory factor analysis (CFA) applied to the two separate forms and the modification indices (MI) suggested by the software were compared. Both forms returned high modification suggestions for adjacent items or items presented near each other. Additionally, it was found that high *χ*^2^ reductions suggested by the MIs in one form resulted in low *χ*^2^ reductions in the other. Lastly, multiple group CFA (mg-CFA) was conducted to determine whether or not measurement invariance was achieved through different item order presentations of the scale. The findings indicate that measurement invariance could not be achieved even at the first stage of analysis. It may specifically be stated that presenting respondents items under the same dimension together ensures empirical findings congruent with theoretical structure.

## Introduction

Data sources have constantly developed and diversified in social sciences, and self-reporting questionnaires are still widely used. There are many factors that may influence the level of information and the validity of the scales used in self-reports. Studies on self-report questionnaires regarding issues caused by responders such as social-desirability (Phillips and Clancy, 1972; Nederhof, 1985; Peltier and Walsh, 1990; King and Bruner, 2000; Larson, 2019), inconsistent or careless responding (Huang et al., 2012; Meade and Craig, 2012; Akbulut, 2015), satisficing (Krosnick et al., 1996; Zhang and Conrad, 2013; Hamby and Taylor, 2016) and their influence on response behavior and scale validity have been widely studied. In addition to these factors, influence stemming from the measurement tool that may affect response behavior may also be significant. One such factor is the instances caused by the presentation of items in different orders. This variation in item order may change the patterns between the responses provided by participants (Schuman and Duncan, 1997). Despite this influence, it may be stated that this matter is largely ignored in the field (Schwarz, 1999).

In situations, where responses by participants differ when the items are presented in different orders, item order effect (also known as question order effect) may be taking place (Tourangeau and Rasinski, 1988). The influence of this effect may also be quite large (Schwarz, 2007). Researchers have been tracking this phenomenon since the 1940’s (Dillman, 2000). It may be stated that research on item order effect as a characteristic of self-report questionnaires has increased in recent years (e.g., Chen, 2010; Schell and Oswald, 2013; Huang and Cornell, 2015, 2016; Weinberg et al., 2018). Unfortunately, the amount of experimental research on this subject appears to be limited (Schuman and Presser, 1996).

Item order effect is especially important for attitude measures. Chen (2010) states that the approach to explaining this phenomenon began with primacy and recency, while in time the literature shifted to anchoring and adjusting. Anchoring and adjusting posits that people tend to anchor based on information initially presented to them, and they derive their plausible estimations through adjustments based on that anchor (Zhao and Linderholm, 2008). Regarding item order effect, the initial responses to items serve as anchors for any subsequent responses (Harrison and McLaughlin, 1993). In other words, anchoring and adjusting occurs when an individual’s stored memory of a context is weak, resulting in prior responses to items serving as anchors, which in turn change the responses given to subsequent items based on these anchors (Chen, 2010).

Despite the fact that awareness on item order effect may be traced back further, it may be stated that research contributing to the literature of the field began in the 1980’s. Since then, studies on item order effect in self-reports have mainly focused on portraying the influence of different item orders on the level of information obtained from the participants. Studies on the comparison of information obtained from presenting items with specific or general statements on the subject first have found broad acceptance in the literature of the field (McFarland, 1981; Strack et al., 1988; Schuman and Presser, 1996; Lasorsa, 2003; Kaplan et al., 2013; Huang and Cornell, 2015, 2016). Since the typical method for determining the item order effect is to apply two different forms with different orders to two groups with similar demographic characteristics (Kaplan et al., 2013), research has mostly been based on this approach. However, when the literature is examined, it can be said that there is a need for research on the impact of item order on self-reports’ psychometric properties, especially on the factorial structure.

### Item Order Effect Studies

In research on item order effect, the focus appears to be on the differentiation obtained as a result of changing the order of a general question and a more specific question on a subject. In a study, which placed either the general question or specific question first through two separate forms, McFarland (1981) found the item order had a low impact on the correlation among items. Schuman and Presser (1996) noted that different results were obtained when two questions on a politically charged subject were asked in differing orders. Strack et al. (1988) used the same method in following years on different subjects, discovering through their research that a differentiation of the presentation order of general-specific questions resulted in a general happiness and dating happiness correlation of 0.16 and 0.55, respectively. In his study of the influence of previously answered items on subsequent items in personality tests, Knowles (1988) created many forms allowing for each item to be presented in every possible position from the beginning to the end of the measure. The findings indicated that the mean score was not influenced by serial position effect, and no interaction was observed between item content and serial position. Additionally, an increase in the reliability values was observed as the item positions moved toward the last positions. The researcher explained this phenomenon by stating that “answering one item leaves a residue that increases the reliability of the next items.” The study concluded that as serial position was advanced, the response consistency of responders increased; that responders continued their initial response tendencies; and that the responses provided were more meaningful predictors of total score, overshadowing the assumption that the measurement tool is independent from the subject measured. In an experimental study, where the key question was asked before and after other items through two separate forms, Lasorsa (2003), observed a 20% variation between responses; the findings indicating that the results obtained may not always be due to individual differences between participants but rather due to the item orders. Chen (2010) studied the influence of item order effects on attitude measures regarding test reliability, item difficulty, item discrimination, test score, test length, reaction time, and person parameters. Findings of Chen (2010) indicated evidence of item order effects on attitude measures. The findings supported the notion that initially presented items may serve as anchors for subsequent questions, as respondents tend to adjust their responses to these subsequent items based on the items presented first. In the first part of their two-part study, Kaplan et al. (2013) determined the mean of the general scale and, subsequently, the strength of the relationship between the general and specific scale changes by rearranging the relative position of the general and specific scales. Similar to the first part, the second part of the study, which used a quasi-experimental design, showed that both the mean of overall satisfaction measure was lower and the magnitude of the specific-general scale relationship was stronger when the general scale preceded the specific scale than in the converse sequence. Bowman and Schuldt (2014) randomized groups of university students found significant difference regarding the order of general and specific questions among the groups, and interpreted this as the influence of item order on item response. Study of Huang and Cornell (2015) also relates to the effect of ordering specific and general items on results. Statistically significant differences were observed in the score averages of the differing forms for each order presented in their experiment. Huang and Cornell (2016) continued their research with a larger and more diverse sample. Regarding the order of the specific and general questions, the test group showed between 20 and 45% differentiation from the control group. Weinberg et al. (2018) conducted a study to evaluate the item order effect from a psychometric validation perspective. They established two forms, one with domain items fixed and general items random, the other with domain items random and general items fixed, and applied these forms to two different groups. The mean values obtained with the fixed domain forms were significantly higher than those obtained from the random domain forms. Additionally, exploratory factor analysis (EFA) and confirmatory factor analysis (CFA) were conducted on both forms separately, with EFA resulting in a one-dimensional structure for both forms, and CFA providing random domain forms with a good fit for one dimensional structure, and a poor fit with fixed domain forms. While this study obtained important and broad findings, the conclusions drawn by the researchers may be skewed due to the demographical imbalances among the groups to which the two forms were presented, with the researchers themselves stating that the findings must be tested with equivalent groups before being presented.

Studies in the literature of the field generally portray findings indicating changes in the correlations between items and mean scores when the general and specific questions are collectively moved based on the characteristic of the measurement. Another significant finding is that items that are responded to first serve as an anchor for those responded to later. Additionally, studies with a small number of but important findings regarding the psychometric properties of the scales show that item order also has an effect on reliability, validity, and item statistics.

In brief, it may be stated that item order effect on different self-report measures has rooted history in the field and continues in popularity today. There are many aspects that may contribute to the field by considering different perspectives and different characteristics of scales regarding item order effects; it is especially emphasized that there is a need for more studies of the influence of item order on the psychometric characteristics of scales, and such studies would be valuable contributions to the literature in the field.

### Current Study

Many factors impact the response patterns of a multi-item self-report questionnaire may be cited (Weinberg et al., 2018). One such factor is the location of the items in the questionnaire. It is assumed that respondents answer adjacent items practically independent from each other and therefore results provide accurate information regarding personal behavior (Bowman and Schuldt, 2014). However, studies on item order effects indicate this situation to be questionable. A study of the literature indicates that studies on item order effect to this date focused on characteristics such as reliability levels, anchor effect, means scores, and item parameters as explained in detail in the previous section. Studies on the influence of item orders or the factorial structures of self-reports are few and are conducted with narrow scopes. Therefore, within the scope of this study, in addition to the descriptives of item order as seen in previous studies, the influence of psychometric properties – especially on the factorial structure of the scale – have also been portrayed; the invariance of the factorial structure in two different forms was tested. To this end, two separate forms were presented to two randomly assigned groups from the same sample. One form was the original scale (fixed order form), while the other form had the items mixed randomly (random order form). The answers to the following research questions were sought:

1. How do the mean scores obtained from the fixed form and random form differ?
2. What are the descriptive values obtained from the CFAs and the reliability values obtained for the fixed order and random order forms?
3. How do the suggested modifications differ for the fixed order and random order forms as a result of the CFAs conducted?
4. Is measurement invariance by form achieved for the fixed order and random order forms?

## Materials and Methods

### Participants

The data of the study were obtained from second to third year undergraduate students studying in seven different departments of an education faculty. 68.5% of the participants were female, while 31.5% were male. 25.4% of the participants were studying in foreign language departments, while 15.9% were in special education, 13.8% in guidance and counseling, 10.7% in primary education, 10% in social sciences, and 8.3% in preschool education. Of the data gathered from 445 students, five were discarded as the participants responded using only one selection throughout the form, and 10 were discarded based on the validation question (see Appendix A), leaving 430 responses remaining with which the study was conducted. After these 430 students were randomly separated into two groups, one was provided with a fixed order form, while the other was given a random order form. In the original state of the scale presented in the fixed order form, the items referring to each of the five dimensions are grouped together. In the random order form, all of the items are randomly presented such that no items referring to the same dimension are presented sequentially. At first, a complete randomization was implemented; however, some items from the same factor were ordered successively when complete randomization was used. Therefore, the locations of those items were changed with another item from a different factor. To answer the research questions and to evaluate the results obtained regarding item order effect, the treatment, and control groups to which the fixed and random order forms were applied should have equivalent demographic characteristics. The chi-square test of independence conducted to ensure the truly randomized assignment of the treatment and control groups resulted in no connection being found between the group (treatment-control) and gender (Pearson *χ*^2^ = 0.41, *p* = 0.52), department (Pearson *χ*^2^ = 0.98, *p* = 0.99) or school year (Pearson *χ*^2^ = 1.76, *p* = 0.62). In other words, the demographic characteristics of the groups to which the fixed and random forms were applied were similar.

### Measures and Procedures

A broad definition of cyberloafing would be employees wasting time at work (Weatherbee, 2010). It may be stated that different types of cyberloafing have been put forth by researchers who based it on different theoretical foundations. Some of these proposals, which stand out include the ego depletion model of self-regulation (Wagner et al., 2012), the theory of planned behavior (Askew et al., 2014), and the theory of interpersonal behavior (Moody and Siponen, 2013). The goal of all these approaches is to explain the nature and predictors of cyberloafing in different settings. However, these studies primarily focused on work-based settings rather than educational environments. The purpose of the cyberloafing scale used in this study is to determine the degree of cyberloafing levels of undergraduate students during lectures.

The scale used within the scope of this study was a five-factor scale consisting of 30 items – namely sharing (nine items), shopping (seven items), real-time updating (five items), accessing online content (five items), and gaming/gambling (four items), originally used to measure participants’ cyberloafing levels, samples of which are included in Appendix A. The original five-factor scale was developed and validated by Akbulut et al. (2016), and used successfully in several studies (e.g., Akbulut et al., 2017; Dursun et al., 2018; Gökçearslan et al., 2018; Kian-Yeik, 2018; Wu et al., 2018; Sivrikova et al., 2019). In the scale’s original state, items within the same dimension were grouped together one after the other. In accordance with the aim of this study, this original form (fixed order form) with items gathered under the same initial dimension and a second form (random order form) in which all items were arranged randomly were created. Since the size of the item order effect may differ based on the demographic characteristics of participants (McFarland, 1981), individuals included in the sample were randomly assigned to the treatment and control groups to ensure that all the demographic characteristics of the groups to which the fixed and random forms were to be applied would be equivalent. Randomization is sufficient to ensure the equivalence of groups in experimental studies as this method ensures the control of all extraneous variables that may influence the research results (Fraenkel and Wallen, 2009). Then, the fixed order form was applied to one of these groups (control group), while the random order form was applied to the other (treatment group). To avoid any bias in responses, the purpose of the study was concealed from the students; the students responded to the items in their forms unaware of the fact that the item orders differed between them. Following data scrubbing procedures, 219 fixed order and 211 random order data sets with a total of 430 data sources were obtained.

### Analysis

The first research question of this study required the use of an independent samples *t*-test to determine the differentiation between the mean values obtained from the fixed order and random order forms. In order to respond to the second research question, first the internal consistency coefficients for the two forms were obtained and secondly, separate CFAs were conducted on both forms to obtain certain item statistics and fit indices.

For the third research question; suggested modifications for improved fit indices, the items from both forms for which these modifications were suggested, and the similarities between the suggested modifications between the two forms were evaluated.

Lastly, to respond to the fourth research question, the invariance of the factorial structure of the scales applied to the treatment and control groups through different item orders was tested. Measurement invariance is achieved when a measurement tool retains the same structure when applied to different groups, or when repeated measurements are conducted on the same groups (Marsh et al., 2015). This process is tested using multiple group CFA (mg-CFA; Dimitrov, 2010; Van De Schoot et al., 2015). Basically, the constancy of the scale in the face of different group characteristics are tested in measurement invariance studies. Within the scope of this study, mg-CFA was utilized to analyze the differentiation in psychometric characteristics among equivalent groups as a result of different item orders on the same scale. In other words, the existence of bias was sought when items of the same factor were applied together or in random order.

The testing of measurement invariance is detailed by Widaman and Reise (1997) in a four-step model. Despite Vandenberg and Lance (2000) proposing an eight-stage approach, the use of this four-stage model is prevalent in the literature (Putnick and Bornstein, 2016). The four stages begin with the least constrained model. The first step, known as configural invariance, freely estimates all parameters in two groups. The second step, called metric invariance (aka weak invariance), forces equal estimation of factor loadings in two groups. The third step, called scalar invariance (aka strong invariance), forces the equal estimation of intercepts in groups. The final stage is called strict invariance and forces the equal estimation of error variances in addition to the previously conducted limitations (Widaman and Reise, 1997). The comparison of models is also conducted step by step. Each step reduces the number of parameters being estimated freely, and the degrees of freedom increase. Each model is nested in the previous model, and the likelihood ratio *χ*^2^ difference test (Bentler and Bonett, 1980) is used to calculate the *χ*^2^ difference between subsequent models, allowing the determination of whether or not the difference in the degrees of freedom between the two models is significant. If no significant difference is found, the limitations in the parameter estimations of that step do not worsen the model-data fit significantly, resulting in the conclusion that measurement invariance is achieved for the step being tested. Therefore, measurement invariance stages are initiated with configural invariance, and if the fit indices indicating the fit of the data with the model that allows for free estimation of all parameters in the groups are good, the next step is conducted. No instance of a breach of univariate normality was encountered in the data distribution. However, the data also showed no multivariate normality, so all CFAs were conducted using maximum likelihood estimation with robust standard errors (MLR).

Regarding software, Jamovi 1.6. (The jamovi project, 2020) was used for the independent samples *t*-test and reliability analyses and Mplus 8.0 (Muthén and Muthén, 1998-2017) was used for the CFAs.

## Results

Firstly, to see whether the order of scales affected descriptive statistics, overall cyberloafing scores of the treatment and control groups were compared. It was observed that the mean in the random order (treatment group; *M* = 74.5, *SD* = 26.1) was significantly higher than the mean in the fixed order (control group; *M* = 69.2, *SD* = 26.9), *t*(428) = 2.07, *p* < 0.05. The effect size for this aforementioned influence was determined as Cohen’s *d* = 0.20.

For the second research question, to portray the influence of item order on the psychometric characteristics of the scale; the reliability coefficients as internal consistency for both forms were calculated for each factor using Cronbach’s *α* and McDonald’s *ω*, and the overall reliability of the scores was calculated using Stratified *α*. For the construct validity findings, the CFA results and other descriptive statistics regarding the items were reported.

A study of the Cronbach *α* and McDonald’s *ω* coefficients obtained in order to determine the reliability through internal consistency (see Table 1) shows that internal consistency coefficients tend to be higher for the fixed order form. In addition, while the Stratified α obtained to evaluate the overall reliability of the scale was slightly higher for the fixed form as with the other subdimensions, the overall reliability obtained for both forms was quite high.

**Table 1:** Internal consistency measures.

Factors	n of items	Fixed order		Random order	
		Cronbach *α*	McDonald’s *ω*	Cronbach *α*	McDonald’s *ω*
Factor 1	9	0.935	0.936	0.926	0.928
Factor 2	7	0.905	0.907	0.869	0.871
Factor 3	5	0.907	0.911	0.896	0.900
Factor 4	5	0.940	0.941	0.917	0.919
Factor 5	4	0.854	0.884	0.825	0.846
Stratified α	30	0.976		0.971	

In the following stage, two separate CFAs were conducted for the five-dimensional structure of the scale on the data obtained with the fixed order and random order forms. The CFAs conducted based on the correlated-traits model resulted in descriptive characteristic values regarding the items for both forms.

The univariate and multivariate normality of the score distribution was tested prior to conducting CFAs. This resulted in the skewness values of the overall scores of the random order and fixed order forms being 0.09 and 0.45, respectively, and kurtosis values of −0.47 and −0.87, respectively. Additionally, the Q-Q plots were analyzed and it was concluded that no instances violating univariate normality in the distribution were present. The multivariate normality of the data was tested using Mardia’s multivariate skewness and kurtosis tests through the MVN package in R (Korkmaz et al., 2014). The lack of multivariate normality was apparent from the significant *p*-values in these tests. Therefore, CFA and mg-CFA were conducted using the MLR estimation method in Mplus 8.0. The correlation values between the dimensions of the scale are presented in Table 2.

**Table 2:** Correlation values between the subdimensions in both forms.

	Shopping		Updating		Accessing		Gaming	
	Fixed	Random	Fixed	Random	Fixed	Random	Fixed	Random
Sharing	0.73^a^	0.70^a^	0.53^a^	0.55^a^	0.78^a^	0.81^a^	0.46^a^	0.40^a^
Shopping			0.60^a^	0.53^a^	0.71^a^	0.77^a^	0.58^a^	0.61^a^
Updating					0.39^a^	0.48^a^	0.36^a^	0.44^a^
Accessing							0.42^a^	0.46^a^

a All correlation coefficients are significant at the value of *p* = 0.001.

Table 2 indicates that the correlations between dimensions were at similar levels for both forms. There were three correlation coefficients that were higher in the fixed form, while the remaining seven correlation coefficients were higher in the random form.

As a result of CFAs conducted for two forms means, SD, factor loadings, *t* values, and residual errors for each item were obtained. A study of these values in Table 3 by matching each item in one form with the corresponding item in the other form showed that the factor loadings of all the items except the last item were above 0.50. The averages for the factor loadings were 0.80 for the fixed order form and 0.76 for the random order form. This difference was not found to be statistically significant [*t*(58) = −1.19, *p* > 0.05]. When the factor loadings of the same items in different forms are analyzed, 19 items had higher factor loading in the fixed form, while 10 items had higher factor loadings in the random form. One item had the same factor loading in both forms.

**Table 3:** Summary of the confirmatory factor analysis (CFA) with different forms.

Factors and items	Fixed order					Random order				
	Mean	SD	F. loading	t value	R. error	Mean	SD	F. loading	t value	R. error
**Factor 1**										
Item 1	3.07	1.31	0.79	27.92	0.38	3.35	1.17	0.80	28.66	0.36
Item 2	2.64	1.23	0.83	35.41	0.31	2.98	1.19	0.74	21.65	0.45
Item 3	2.36	1.33	0.84	38.28	0.29	2.58	1.34	0.74	22.13	0.44
Item 4	3.22	1.31	0.77	26.10	0.40	3.52	1.24	0.81	29.21	0.34
Item 5	2.00	1.04	0.71	19.77	0.50	2.23	1.17	0.80	26.36	0.36
Item 6	2.02	1.14	0.77	25.50	0.41	2.29	1.29	0.89	43.76	0.21
Item 7	2.20	1.26	0.80	29.58	0.36	2.48	1.29	0.58	11.62	0.66
Item 8	3.66	1.11	0.74	22.50	0.45	3.98	1.12	0.55	1.89	0.69
Item 9	3.09	1.50	0.79	27.72	0.38	3.14	1.48	0.66	15.74	0.56
**Factor 2**										
Item 10	2.39	1.39	0.88	42.21	0.23	2.65	1.40	0.82	33.26	0.32
Item 11	1.70	1.14	0.75	23.03	0.44	1.45	0.96	0.73	2.41	0.46
Item 12	2.63	1.38	0.85	35.53	0.28	2.87	1.28	0.81	29.83	0.35
Item 13	1.83	1.22	0.78	25.76	0.40	2.11	1.24	0.81	3.86	0.33
Item 14	2.60	1.39	0.75	23.38	0.43	2.93	1.39	0.79	27.28	0.37
Item 15	1.81	1.24	0.73	2.66	0.46	1.95	1.19	0.86	38.13	0.26
Item 16	1.58	1.06	0.57	11.73	0.67	1.61	1.03	0.91	6.59	0.17
**Factor 3**										
Item 17	1.65	1.13	0.80	28.50	0.35	1.92	1.26	0.5	9.08	0.74
Item 18	2.53	1.48	0.82	31.23	0.33	2.75	1.44	0.69	17.71	0.52
Item 19	1.92	1.30	0.89	46.65	0.22	2.08	1.35	0.84	37.37	0.29
Item 20	2.14	1.42	0.88	44.01	0.22	2.42	1.45	0.86	36.70	0.26
Item 21	1.67	1.07	0.71	18.93	0.50	1.82	1.09	0.71	18.53	0.50
**Factor 4**										
Item 22	2.68	1.61	0.81	33.79	0.34	2.93	1.59	0.75	22.94	0.43
Item 23	3.05	1.62	0.96	129.0	0.08	3.21	1.60	0.92	64.44	0.16
Item 24	2.93	1.74	0.97	145.7	0.07	3.10	1.70	0.87	41.31	0.24
Item 25	2.32	1.47	0.71	2.57	0.49	2.28	1.39	0.93	49.42	0.14
Item 26	3.04	1.50	0.86	46.34	0.26	3.24	1.36	0.63	13.76	0.60
**Factor 5**										
Item 27	1.40	1.02	0.97	11.76	0.07	1.52	1.09	0.89	46.48	0.20
Item 28	1.35	0.96	0.98	116.25	0.05	1.43	0.97	0.75	22.53	0.44
Item 29	1.79	1.25	0.75	24.08	0.44	1.91	1.30	0.66	15.29	0.56
Item 30	1.98	1.26	0.44	7.80	0.81	2.10	1.35	0.53	9.80	0.72

When the CFAs conducted for the random order and fixed order forms are studied (see Table 4), the fit indices obtained for the fixed order form were found to be slightly better than those of the random order form, and very close to the acceptable fit values.

**Table 4:** Fit indices for the CFAs without any modifications.

Fit criteria	Fixed order	Random order	Acceptable	Reference
RMSEA	0.083 (0.076–0.089)	0.92 (0.086–0.098)	<0.08	Hooper et al., 2008
CFI	0.87	0.83	>0.90	Hu and Bentler, 1999
TLI	0.86	0.81	>0.90	Schumacker and Lomax, 1996
SRMR	0.09	0.110	<0.08	Hu and Bentler, 1999

The third research question is directed at portraying whether or not the suggested modifications to improve the model for both forms as a result of the CFAs are influenced by item order. To this end, the modification indices (MI) proposed for both forms that ensured the largest *χ*^2^ reduction were compared (see Table 5). One of the fundamental assumptions of structural equation modeling is that there should not be a relationship between the residuals of observed variables (Kline, 2011). Therefore, considering modification applications conflict with this fundamental assumption, it may be stated that only a limited amount of modification that can be theoretically explained and ensures a large decrease in *χ*^2^ in accordance with the parsimony principle should be applied.

**Table 5:** Suggested modifications with the highest *χ*^2^ reductions in both forms.

Form	Suggested largest MI (*χ*^2^ > 30)	Suggested *χ*^2^ reduction	Order of the items in the other form	Suggested *χ*^2^ reduction for the other form
Fixed	item 10-item 12	84.6	item 3-item 22	-
Fixed	item 1-item 2	48.0	item 2-item 4	-
Fixed	item 23-item 24	43.8	item 19-item 23	-
Random	item 27-item 28	58.0	item 6-item 23	-
Random	item 1-item 2	53.1	item 2-item 13	-

In Table 5, the first column indicates which form the items suggested for modification are in, the second column indicates order of the items for which modifications were suggested, and the third column indicates the *χ*^2^ reduction if the modification is applied. Based on the parsimony principle, suggested modifications that would reduce the *χ*^2^ value 30 or more for both forms were reported. The fourth column indicates corresponding item orders to the respective MI in the other form, while the last column indicates how much of a *χ*^2^ reduction is caused for the suggested MI for these items.

The values in the Table 5 show that defining the relationship between the residual errors of items 10 and 12 in the fixed order form resulted in a large *χ*^2^ reduction of 84.6, while these items were numbered 3 and 22 in the random order form, and the suggested modification of the related items in this form resulted in a *χ*^2^ reduction of less than 10. Similarly, the *χ*^2^ reduction for items 1 and 2, and 23 and 24 in the fixed order form was found to be under 10 regarding their corresponding items in the random order form. An analysis of the corresponding modifications in the fixed order form of the high modifications suggested for the random order form resulted in a similar situation. As such, while the high *χ*^2^ reduction for the suggested modifications for items 27 and 28 in the random order form resulted in a value of 58.0, the same items in the fixed order form (items 6 and 23) resulted in a *χ*^2^ reduction of less than 10. Similarly, the suggested modification for items 1 and 2 in the random order form was 53.1, while the same items in the fixed order form, at numbers 2 and 13, resulted in a suggested modification under 10. In brief, the large modifications suggested were for items that were either successive or at most two items apart from each other in both forms, and changing the orders of these items in their respective forms also changes the suggested modifications.

To answer the fourth research question, mg-CFA was conducted to test measurement invariance. Despite there being a consensus in the literature regarding the four stages of measurement invariance, some researchers have stated that the final, strict invariance stage is an unnecessary test. This is supported by the fact that error variances are no longer part of the latent variable and therefore inconsequential when comparing latent variable means (Vandenberg and Lance, 2000). This results in most researchers excluding the final stage (Putnick and Bornstein, 2016). Therefore, the final stage was omitted in this study, and configural, metric, and scalar invariance were tested in stages. To this end, version 8.0 of Mplus, which has a syntax that allows for the simultaneous execution of all three stages (Şen, 2020), was used. A study of the fit indices (see Table 6), used to evaluate whether or not configural invariance was achieved, shows that none of these indices reach the acceptable cut-off values. Based on this finding, it was observed that the model-data fit obtained was poor, therefore not even configural invariance, the first stage of measurement invariance, and was achieved. In other words, the two different forms with different item orders may result in different evaluations-understandings of the scale by two equivalent groups, therefore causing bias.

**Table 6:** Fit indices for the configural invariance model.

Fit criteria	Values	Acceptable	Reference
RMSEA	0.087 (0.083–0.092)	<0.08	Hooper et al., 2008
CFI	0.85	>0.90	Hu and Bentler, 1999
TLI	0.84	>0.90	Schumacker and Lomax, 1996
SRMR	0.10	<0.08	Hu and Bentler, 1999

## Discussion

Many situations may influence the information level obtained from self-report questionnaires. One such instance is item order effect, in which respondents’ response behaviors change due to items comprising a scale being presented in different orders. Within the scope of this study, a multidimensional scale developed for determining individuals’ cyberloafing levels with well-established factorial structure was utilized. Two forms were created; one form was the original scale with all items of a factor presented together, while the other form presented items randomly. Two groups were established randomly from a sample, and the fixed order form was presented to the control group, while the random order form was presented to the treatment group. The goal here was to determine whether or not the descriptive statistics and response patterns of the scale were influenced by item order. Initially, the means were compared using an independent samples *t*-test, and the means obtained from the random order form was found to be significantly different than that of the fixed order form. This was followed by separate CFAs for both forms, and while the value obtained for the fixed form was slightly better, both scales required modification for the model-data fit to reach acceptable levels. Considering the modifications conducted to both forms, the modifications proposed for the fixed form can be theoretically explained, while those of the random form cannot. Additionally, the large MIs suggested for each form were analyzed for their counterparts in the other forms, and it was found that the respective counterparts of the high modifications were actually very low in the other form. In the final step, mg-CFA was conducted to analyze the influence of item order effect on measurement invariance. The results indicate configural invariance, which is the first step in measurement invariance, which allows the free estimation of all parameters in both groups, yielded fit indices regarding the model-data fit below acceptable values. This result led to the conclusion that measurement invariance could not be achieved, and item order caused a bias regarding the factorial structure of the scale.

For a long time in the use of self-reports, respondents’ responses to an item were thought to be perfectly isolated from adjacent items. However, many studies have disproven this assumption (Bowman and Schuldt, 2014). While the purpose of this study is broader in scope than item order studies in the literature, it may be stated that it showed similarity regarding descriptive statistics being influenced by item order. Many studies, as with this study, have shown that mean scores obtained with equivalent groups being presented with different item orders in their self-reports differentiate (Schuman and Presser, 1996; Lasorsa, 2003; Kaplan et al., 2013; Saeki et al., 2013; Huang and Cornell, 2015, 2016; Shorey et al., 2016). In their study with a similar scope to the current study, Weinberg et al. (2018) separately conducted EFA and CFA on the data they obtained from forms they presented in different manners to two groups. They found that while the modifications proposed for domain random order were not explainable, the modifications proposed for the other form were theoretically explainable. However, the fact that the groups in that study were not established with random assignment should not be disregarded.

One of the fundamental goals of this study was to determine whether the adjacent presentation of items theoretically under the same dimension created a bias regarding the factorial structure of the scale. To this end, the separate CFAs conducted on the two data sets resulted in the high modifications for both groups being very different from each other. So much so that applying the suggested MIs for the fixed order form to the random order form resulted in extremely low reductions in *χ*^2^. Similarly, the large MIs proposed for the random order form had very low counterparts in the fixed order form. Additionally, the three large MIs suggested for the fixed order form are all theoretically explainable, while the two large MIs suggested for the random order form do not have a theoretical basis. Considering the suggested modifications for both forms resulting in a very high *χ*^2^ reduction are for either adjacent or with one item in between, it is understandable that the only theoretically explainable modifications are within the fixed order form, in which items belonging to the same factor are presented sequentially. Therefore, these findings may be interpreted as MIs with *χ*^2^ reductions that may cause significantly improved model-data fits being a result of the influence of item order rather than any theoretical commonalities between the items. Thus, it may be stated that the close proximity of items has a significant effect on the response behaviors of respondents.

The findings obtained in the final stage of the study indicate that measurement invariance cannot be achieved even in the first stage due to configural invariance not yielding acceptable model-data fit values. As such, it may be stated that presenting scale items in different orders influences factorial structure, causing significant bias.

These findings portray the effect of item order on factorial structure. It may specifically be stated that presenting respondents items under the same dimension together ensures empirical findings congruent with theoretical structure. As such, the findings provide the opportunity to propose significant recommendations for both theoretical and practical applications. It may be stated that since the proposed modifications differentiate based on item order rather than theoretical basis, the local independence assumption is overshadowed. In practice, however, it is believed that in order to prevent the factorial structure being influenced by items of the same dimension being presented together, this situation must be taken into consideration when ordering items of multidimensional measures and the highest possible randomization is considered to be beneficial. Specifically, a significant recommendation derived from the findings of this study would be that researchers avoid presenting items from the same dimension together in order to achieve the expected theoretical structure during scale development. In the instance that items from the same dimension do end up one after the other, the findings of this study may be beneficial when defining the MIs proposed for these items.

### Limitations and Recommendations for Future Research

The sample of this study consisted of students at an education faculty of a university. Similar studies on this subject may be conducted with a broader sample or individuals with different age groups. Another limitation of this study was that it was executed using the cyberloafing scale. The characteristics of the scale may have an effect on the results. Similar studies on different psychological constructs may be beneficial in increasing the generalizability of the findings of this study.

Within the scope of this study, a scale that was developed earlier and which has had its structural validity confirmed with different samples was used. In future research, the approach used in this study may be applied to the scale development phase. A scale foreseen to be multidimensional may benefit from EFA of the data regarding the combined presentation of items expected to be under the same dimension theoretically and the random order presentation of these items. Another interesting study would be the effect of many random order forms applied to equivalent groups on dimensionality through a scale expected to be one dimensional using a similar research design. Weinberg et al. (2018) proposed a similar recommendation, especially emphasizing the research of situations emerging from the systematic manipulation of item order randomization in the future.

This study could also be conducted on the same groups by administering two different forms. Such a study could analyze the influence of item order on the data obtained from the same group by dividing the sample into two at random, followed by first providing one group with the fixed form then the random order form, and providing the other group with the fixed form and random order form to conduct this analysis.

## Data Availability Statement

The dataset for this study is available at https://osf.io/qesm4/.

## Ethics Statement

Ethical review and approval was not required for the study on human participants in accordance with the local legislation and institutional requirements. Written informed consent to participate in this study was provided by the participants.

## Author Contributions

The author confirms being the sole contributor of this work and has approved it for publication.

### Conflict of Interest

The author declares that the research was conducted in the absence of any commercial or financial relationships that could be construed as a potential conflict of interest.

## Appendix A

Sample Items and Validation Question

**Table:**

Subdimension	Sample item
Sharing	I like posts that are interesting
Shopping	I check job advertisements
Real-time updating	I comment on trending topics
Accessing online content	I download applications I need
Gaming/Gambling	I check online sport sites

Validation question: I responded all the items in this questionnaire correctly.
